# 3D Numerical Modeling of Laser Assisted Tape Winding Process of Composite Pressure Vessels and Pipes—Effect of Winding Angle, Mandrel Curvature and Tape Width

**DOI:** 10.3390/ma13112449

**Published:** 2020-05-27

**Authors:** Amin Zaami, Ismet Baran, Ton C. Bor, Remko Akkerman

**Affiliations:** Faculty of Engineering Technology, Chair of Production Technology, University of Twente, 7500AE Enschede, The Netherlands; a.zaami@utwente.nl (A.Z.); t.c.bor@utwente.nl (T.C.B.); r.akkerman@utwente.nl (R.A.)

**Keywords:** laser assisted tape winding/placement, simulation model, thermoplastic composites, helical winding/placement

## Abstract

Advanced thermoplastic composites manufacturing using laser assisted tape placement or winding (LATP/LATW) is a challenging task as monitoring and predicting nip point (bonding) temperature are difficult especially on curved surfaces. A comprehensive numerical analysis of the heat flux and temperature distribution near the nip point is carried out in this paper for helical winding of fiber reinforced thermoplastic tapes on a cylindrically shaped mandrel. An optical ray-tracing technique is coupled with a numerical heat transfer model in the process simulation tool. The developed optical-thermal model predictions were compared with experimental data available in literature to validate its effectiveness. The influences of winding/placement angle, mandrel curvature and tape width on the incident angles, the laser absorbed intensity, and the process temperature distribution are studied extensively using the validated model. Winding/placement angle has a considerable effect on the temperature distribution. Increase in winding angle results in a higher temperature for tape due to more reflections coming from the substrate. On the other hand, substrate temperature decreases as the winding angle increases due to a decrease in the laser incident angles based on the local surface curvature. An increase in mandrel curvature results in higher nip point temperatures for substrate and lower one for tape. Different mandrel sizes for 90° placement path do not have a strong effect on the substrate process temperature as for other winding angles because of less curvature change of the corresponding irradiated area. Tape width causes local temperature variations at the edges of the tape/substrate. In order to obtain the desired process temeprature during LATW or LATP processes, the laser intensity distribution on the tape and substrate surfaces should be regulated.

## 1. Introduction

Laser assisted tape placement (LATP) is an automated manufacturing process to produce fiber reinforced thermoplastic composite structures. The out-of-autoclave potential of the LATP method together with its automation possibilities make it attractive for aerospace and automotive industries. The LATP method is also suitable for winding of prepreg fiber reinforced thermoplastic tapes on a cylindrical liner/mandrel via the laser assisted tape winding (LATW) process to manufacture composite parts such as pipes for oil and gas industry or hydrogen storage pressure vessels for the transportation industry.

**Figure 1:**
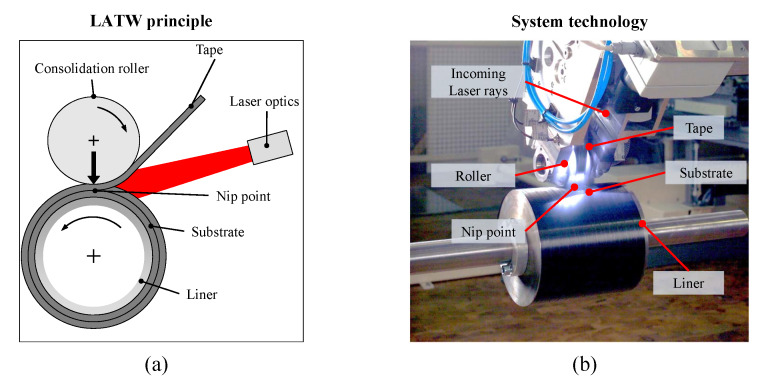
Description of the laser assisted tape placement or winding (LATP/LATW) method and its elements including laser optics, substrate, tape, roller and mandrel. The hoop winding/placement process where thermoplastic tape and substrate are preheated by the laser source and the tape is pressed on the substrate at the nip point location through a consolidation roller are shown as: (**a**) Principle, (**b**) System technology (detailed view on the experimental set-up employed at Fraunhofer IPT, Aachen, Germany).

Figure 1a schematically shows the LATP/LATW process method in which the fiber reinforced thermoplastic tape is placed onto a mandrel and/or a composite substrate. Figure 1b illustrates an image of a hoop winding/placement on a metal mandrel. The incoming tape and substrate are heated using a laser and bonded under the application of a pressure exerted by a compaction roller. An in-situ consolidation can be achieved by a proper adjustment of the applied heat, pressure and process speed. The temperature of the nip point at which the tape and substrate are bonded plays a critical role together with the consolidation pressure and time for having proper intermolecular diffusion and healing of the thermoplastic matrix. The importance of process parameters on the mechanical performance and bonding quality was shown extensively in References [1,2,3,4,5,6]. A proper understanding and description of the laser irradiation and reflection which determine the temperature distribution near the nip point is therefore crucial for in-situ consolidation using LATP and LATW processes. The role of absorption and reflection of the laser irradiation by the material was also illustrated in Reference [7] during a powder bed fusion process. The composite tape has an angle-dependent laser absorption and reflection which make the LATP and LATW processes very challenging to control especially for manufacturing on curved surfaces. In addition, the variation in material, geometrical and process parameters causes a variation in the bonding temperature [8]. Therefore, maintaining the nip point temperature at desired values is needed to obtain a proper consolidation and adhesion between the subsequent layers [9].

Several studies in literature investigated the temperature evolution and distribution during LATP and LATW processes experimentally and computationally. Thermal models were developed in [10,11,12,13,14,15,16,17,18,19,20] by assuming a uniform heat flux exerted on the substrate and tape for the sake of simplicity in numerical process modeling. An analytical thermal solution of the LATP process on a flat tooling was developed in Reference [10] in which the through-thickness temperature distribution was investigated at high process speeds. A significant drop in temperature prior to the nip point was found in [11] due to the presence of a shaded region originated from the roller geometry. The effects of variation in material properties, thickness and fiber volume fraction on the substrate temperature were studied statistically in Reference [21] for the LATW of AS4/PEEK composites. A two dimensional (2D) thermal model was coupled with a crystallization model in References [14,15] and the influence of process parameters on the crystallization was studied. The sensitivity of the temperature with respect to the winding/placement speed and laser power was investigated in Reference [13] for tape laying of continuous reinforced carbon fiber (CF)/PEEK laminates. The effects of processing parameters on processability in on-line consolidation of thermoplastic composites was studied in Reference [17] for tape-laying and filament-winding processes employing anisotropic thermal analyses. The results showed that the heater size, preheating conditions and tow thickness affected the processing window significantly. The analysis in Reference [12] predicted the temperature distribution along the length and through the thickness of the flat composite laminate during a tape laying process. The tape lay-up processing speed, nozzle exit temperature and cooling rate were found to be the major process variables. The effects of preheating the consolidated flat laminate, temperature distributions and thermal histories were investigated for varying consolidation speeds and the overall feasibility of the proposed process was discussed in Reference [16].

The absorption and reflection behavior of the composite material during the LATP and LATW processes were taken into account in References [6,22,23]. More specifically, dedicated optical models which determined the heat flux distribution as an output variable were developed in order to couple it with thermal models for having a better prediction of the temperature distribution near the nip point. A specular reflection model was used in Reference [6] for LATP on flat surfaces by incorporating a 2D ray-tracing method which was combined with a 1D thermal model in the through-thickness direction. The anisotropic optical behavior of the tape and substrate was described using a bidirectional reflectance distribution function (BRDF) in Reference [22] for the circular winding/placement cases. A micro half cylinder (MHC) approach was presented in Reference [23] to simulate the laser irradiance scatter via a commercial software (OptiCAD 10) in the LATP process on a flat substrate or tool. The temperature predictions using the coupled optical-thermal process models for the LATP in Reference [6] revealed that the incident flux distribution on the substrate decreased rapidly near the nip point due to the shadowing effect of the roller. The same optical model developed in Reference [23] was used in Reference [24] to predict the laser power distribution in the 2D thermal analysis of LATP on flat surfaces using the commercial finite element software package ANSYS. The variations of tape and substrate temperature using different assumptions for the laser divergence modeling were reported.

Besides the thermal phenomena investigated for the LATP processes on flat tools or substrates, the LATW of relatively simple non-flat geometries was studied for the case of ring winding in References [25,26]. The through-thickness temperature evolution in the substrate made of CF/PEEK was quantified in Reference [25]. The BRDF was used in a 3D ray-tracing model coupled with a 1D heat transfer model in Reference [26] to predict the temperature distribution for different process parameters in LATW of circular CF/PEEK composites. The influence of different laser power distributions on the nip point temperature was studied in Reference [26]. Although there have been several studies carried out to describe the temperature distribution in the LATP and LATW process of fiber reinforced thermoplastic composites, a critical assessment of the helical winding/placement on cylindrical mandrels, for example, for composite pipe and pressure vessel manufacturing, has not been considered. The laser irradiation and reflection determining the heat flux distribution on curved surfaces inherently contains a complexity due to the variation in the incident angle of the laser light with respect to the winding/placement angle and curved surface geometry [27,28]. In addition, the interaction of rays and reflections with the substrate and tape can vary as the tape/substrate width changes. In Reference [29], it was clearly stated that overheating of the tape or substrate affected the intralaminar void content, intralaminar bond strength and residual stress negatively. Besides, an insufficient bonding may take place if the temperatures near the nip point are below those within the processing window. These aspects need to be analyzed comprehensively in order to develop optimum process conditions to maintain a constant nip point temperature during the process.

In the present work, the influence of the winding/placement angle, the mandrel diameter and the tape width on the temperature distribution near the nip point is numerically investigated for the helical LATP/LATW process of fiber reinforced thermoplastic composites as a part of the EU funded ambliFibre project. The optical and thermal models are part of the comprehensive OTOM (Optical Thermal Optimization Model) simulation tool developed by using MATLAB at the University of Twente for the simulation of LATW/LATP processes. A generic 3D optical model is developed based on a ray-tracing technique incorporating the varying incident angles due to the helical path curvature on the mandrel. The predicted heat flux distributions obtained from the optical model are used in a 2D and 3D numerical thermal model for tape and substrate, respectively, in order to predict the temperature distributions. The coupled optical-thermal process model is validated by comparing the predicted temperature evolution with available literature data for the LATP process using a flat tooling. The validated model is subsequently applied to the LATP/LATW process on non-flat tooling and the behavior of the absorbed laser power intensity distribution is described based on the surface curvature of the tooling, the winding/placement angle and the tape width. The variations in the incident angle of incoming rays and reflections on the substrate and tape are analyzed quantitatively. The characteristics of the temperature distribution as well as the total absorbed energy from the direct laser hit and reflections are studied for helical winding/placement of tapes on different curved surfaces, that is, different mandrel diameters.

## 2. Optical Model

**Figure 2:**
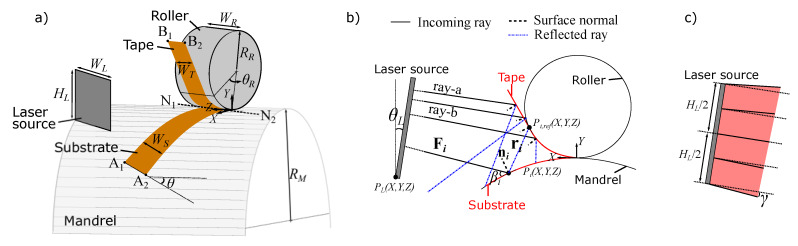
(**a**) Geometry of the parametric 3D optical model. (**b**) Schematic of ray-tracing approach with incoming ray and reflection for tape (ray-a), substrate ($F_{i}$) and roller (ray-b). (**c**) Schematic of the laser divergence angle γ which is maximum at the edges and linearly decreases to 0° at the center of laser source.

A parametric 3D optical model based on the ray-tracing approach was developed for LATP and LATW processes using an in-house code written in MATLAB. A schematic view of the model geometry is seen in Figure 2a. A global coordinate system denoted as X,Y and *Z* was used in the optical model. Here, $W_{R}$ and $R_{R}$ were the roller width and radius, respectively, $W_{L}$ and $H_{L}$ were the width and height of the laser source defined as a plane, respectively. A top-hat laser power distribution with a divergence angle of γ was considered [30]. The winding/placement angle θ, the substrate width $W_{S}$ and the tape width $W_{T}$ were assumed to be constant during the manufacturing process.

The winding/placement direction along which the roller moved to deposit the unidirectional tape on the substrate was defined by θ. The substrate was therefore oriented on the mandrel (tooling) in the direction defined by the θ. The substrate domain on the cylindrical mandrel was calculated as a function of mandrel radius $R_{M}$, $W_{S}$ and θ using a closed form solution:

(1) $$\begin{array}{cc} \; & X_{path}=R_{M}\;\sin\left(\eta \frac{\sin(\theta )}{R_{M}}\right)+R_{M}\;\sin\left(\xi \frac{\cos(\theta )}{R_{M}}\right),\;0<\eta <W_{S},\;0<\xi <L_{S} \end{array}$$

(2) $$\begin{array}{cc} \; & Y_{path}=R_{M}\;\cos\left(\eta \frac{\sin(\theta )}{R_{M}}\right)+R_{M}\;\cos\left(\xi \;\frac{\cos(\theta )}{R_{M}}\right),\;0<\eta <W_{S},\;0<\xi <L_{S} \end{array}$$

(3) $$\begin{array}{cc} \; & Z_{path}=\eta \;\cos\left(\theta \right)+\xi \;\sin\left(\theta \right),\;0<\eta <W_{S},\;0<\xi <L_{S} \end{array}$$

where $L_{S}$ was the substrate length, ξ and η were variables of substrate width and length for defining curved substrate locations denoted as $X_{path},Y_{path},Z_{path}$ (based on right-hand axis system of the mandrel at its center). Executing these equations in the optical model, different substrate orientations on the mandrel can be generated. The contact region between the tape and roller was defined with the angle $\theta_{R}$. A schematic representation of the generic 3D ray-tracing approach is seen in Figure 2b. The position of the laser source, that is, the location $P_{L}\left(X,Y,Z\right)$ and orientation $\theta_{L}$, was defined with respect to the nip line N_1_-N_2_ at which the origin of the global optical coordinate system was located. A total of $N_{ray}$ rays was used to model the laser power distribution. Each incoming laser ray was defined as a 3D line which is represented as $F_{i}$ for the *i*th ray in Figure 2b. The intersection point of $F_{i}$ with a 3D parametric surface, for example, $P_{i}\left(X,Y,Z\right)$, was determined analytically and the corresponding surface normal $n_{i}$ and the incident angle $\beta_{i}$ were calculated. The 3D reflected ray $r_{i}$ was obtained using the expression $r_{i}=2\left(F_{i}\cdot n_{i}\right)n_{i}-F_{i}$. Similarly, the intersection point of $r_{i}$ with another parametric surface, for example, $P_{i,ref}\left(X,Y,Z\right)$, together with $n_{i,ref}$ and $\beta_{i,ref}$ were calculated. The defined rays can collide with multiple surfaces, for example, the tape (indicated as ray-a in Figure 2b), the roller (ray-b), the substrate and the mandrel in the optical model.

**Figure 3:**
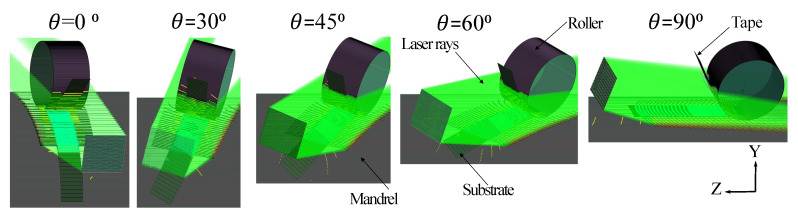
Different placement angles on the reference mandrel. In this picture, the green lines are rays, blue region on mandrel is the substrate, and the blue region attached to the roller is the tape (for interpretation of the references to color in this figure legend, the reader is referred to the web version of this article).

**Figure 4:**
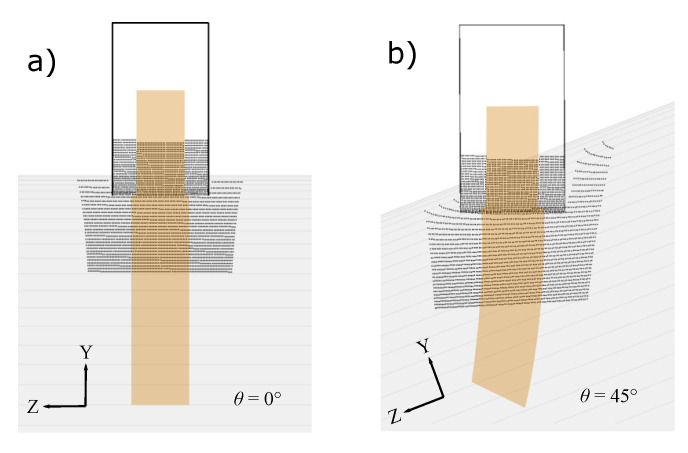

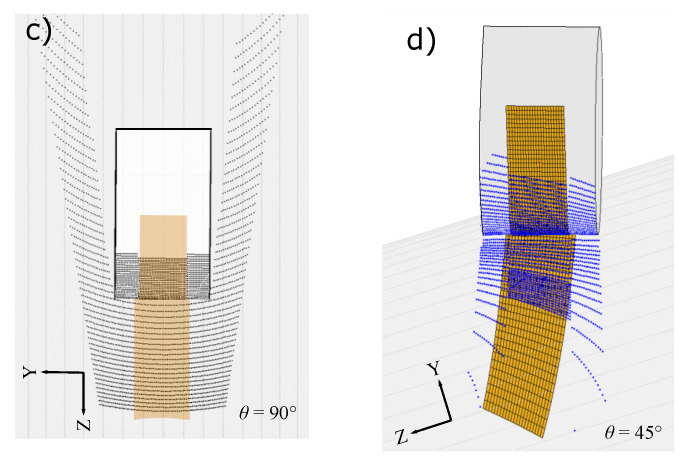
Intersection of the incoming rays with the mandrel, the tape, the substrate and roller for (**a**) θ=0°, (**b**) θ=45° and (**c**) θ=90°. (**d**) The corresponding intersection of the reflected rays for θ=45°.

A representation of the simulation model showing the placement angles of 0°, 30°, 45°, 60°and 90° is shown in Figure 3. In all cases, the relative orientation and location of the laser cross-section plane toward the nip-point were remained constant. However, the relative locations of the points on substrate toward the laser plane were changed due to the curvature. This effect can strongly change the absorbed energy intensity and the process temperature as a result of changes in the irradiated area and the irradiation angle. The intersection points of incoming rays with the 3D surfaces for θ=0°, θ=45° and θ=90° are illustrated in Figure 4a–c, respectively. It is seen that the curvature of the mandrel and the laser divergence were taken into account in the developed model. The corresponding intersection points of the reflected rays with substrate, tape, mandrel and roller are shown in Figure 4d for θ=45°, as an example. The incoming rays were assumed to reflect specularly in the current model for the sake of simplicity (ease of analysis) and computational time.

**Figure 5:**
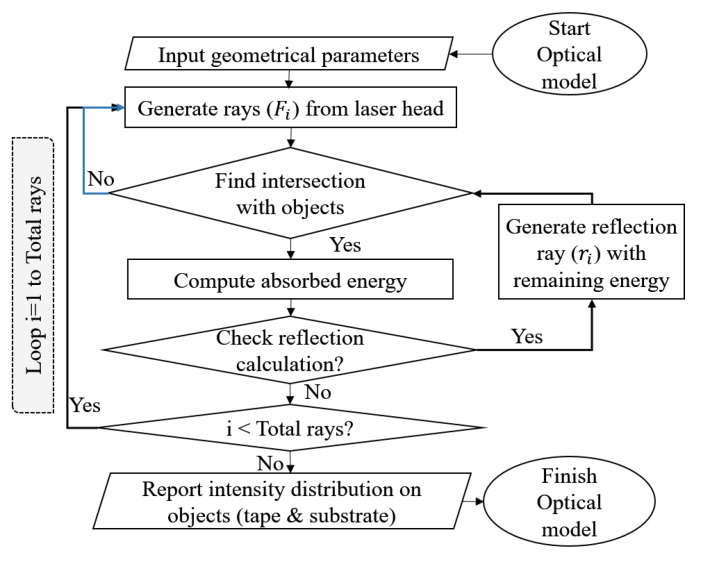
Flowchart of the optical model.

The rays were discretized uniformly along the length and width of the laser source plane. The irradiation power distribution of the *i*th ray, $\Phi_{i}$, was calculated by dividing the total laser power $P_{laser}$ by $N_{ray}$. The conservation of the energy was maintained by considering:

(4) $$\Phi_{i}=\phi_{i,a}+\phi_{i,r}$$

where $\phi_{i,a}$ and $\phi_{i,r}$ were the the absorbed and reflected energy at the intersection point $P_{i}\left(X,Y,Z\right)$, respectively. Here, the transmitted energy was neglected. $\phi_{i,a}$ was calculated using the following expression:

(5) $$\phi_{i,a}=\Phi_{i}F_{i}\left(n_{i},\beta_{i}\right)$$

where $F_{i}\left(n_{i},\beta_{i}\right)$ was the fraction of total energy calculated by using the unpolarized Fresnel equations [22,31] and $n_{i}$ was the refractive index. Similarly, the absorption of the reflected energy $\phi_{i,r}$ at point $P_{i,ref}\left(X,Y,Z\right)$ was calculated using $\phi_{i,a,ref}=\phi_{i,r}F_{i,ref}\left(n_{i,ref},\beta_{i,ref}\right)$. This approach was successively continued for the total number of rays and reflections until all intersection points of the incoming rays and corresponding energy were calculated. Note that only one reflection was considered in the optical model because the energy carried by the second and following reflected rays were estimated as less than 5% of the energy of the incoming ray [26]. The total number of rays used in the optical model was 32 k (160 along the width and 200 along the height of the laser source plane). The flowchart of the described optical model is represented in Figure 5.

## 3. Thermal Model

**Figure 6:**
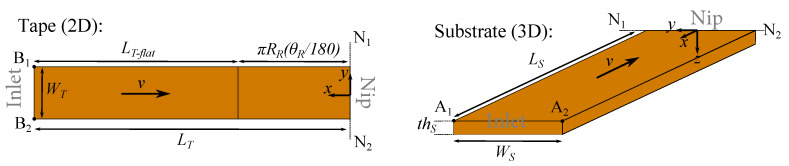
Schematic view of the thermal domains for tape and substrate which were unfolded from the optical domain defined in Figure 2.

A local thermal model was developed by employing the control volume based finite difference technique in order to predict the temperature distribution prior to the nip line (N_1_-N_2_ in Figure 2a). The generic tape and substrate geometries defined in the optical model were unfolded to the computational domains in the thermal model as shown in Figure 6. A local coordinate system denoted as x,y and *z* was employed in the thermal model. A structured control volume based mesh was therefore defined in the local (x,y,z) coordinate system. The thickness of the incoming tape is usually very thin as compared to the substrate thickness which yields a uniform temperature distribution in the through-thickness direction for the tape. Therefore, 2D and 3D heat conduction models were considered for generic purposes for the tape and substrate, respectively. The advection term in the heat transfer equation was implemented using a Eulerian frame work. The corresponding governing equations given in Equations (6) and (7) for the tape and substrate, respectively, were solved using an upwind implicit scheme as described in Reference [32].

(6) $$\rho C_{p}\left(v\frac{\partial T}{\partial x}\right)=k_{x}\left(\frac{\partial^{2}T}{\partial x^{2}}\right)+k_{y}\left(\frac{\partial^{2}T}{\partial y^{2}}\right),\;\;\mathrm{Tape}$$

(7) $$\rho C_{p}\left(v\frac{\partial T}{\partial x}\right)=k_{x}\left(\frac{\partial^{2}T}{\partial x^{2}}\right)+k_{y}\left(\frac{\partial^{2}T}{\partial y^{2}}\right)+k_{z}\left(\frac{\partial^{2}T}{\partial z^{2}}\right).\;\;\mathrm{Substrate}$$

In Equations (6) and (7), *T* was the temperature, *v* was the winding/placement velocity of the tape showing the material movement toward the nip line, $C_{p}$ was the specific heat, ρ was the density, x,y,z represent the local spatial locations, $k_{x},k_{y},k_{z}$ were the coefficient of thermal conductivity of the composite material in *x*-, *y*- and *z*-direction, respectively. Thermal properties were assumed to be independent of the temperature in the current research as also considered in References [2,6]. In order to show the finite difference implementation, the equation in Equation (7) as a general 3D case was discretized for an exemplary interior control volume denoted as i,j,k along the *x*-, *y*- and *z*-direction, respectively in Equation (8).

(8) $$\begin{array}{c} \begin{array}{c} \rho C_{p}\left(v\frac{T_{i,j,k}-T_{i-1,j,k}}{\Delta x}\right)=k_{x}\left(\frac{T_{i+1,j,k}-2T_{i,j,k}+T_{i-1,j,k}}{\Delta x^{2}}\right)+\\ k_{y}\left(\frac{T_{i,j+1,k}-2T_{i,j,k}+T_{i,j-1,k}}{\Delta y^{2}}\right)+k_{z}\left(\frac{T_{i,j,k+1}-2T_{i,j,k}+T_{i,j,k-1}}{\Delta z^{2}}\right) \end{array} \end{array}$$

where Δx, Δy and Δz were the distance between the control volumes in *x*-, *y*- and *z*-direction, respectively. The formulation in Equation (8) was transformed to a matrix notation and assembled for *m* number of total control volumes which can be represented as:

(9) [K]{T}={Q}

where [K] was the m×m thermal conductivity matrix, {T} was the m×1 temperature vector and {Q} was the m×1 thermal load vector. The following boundary conditions were employed in the thermal domains as illustrated in Figure 6:

Tape (2D):

(10) $$\begin{array}{cc} \; & \pm \left(k_{x}\frac{\partial T}{\partial x}+k_{y}\frac{\partial T}{\partial y}\right)=q_{ir}+h_{air}\left(T-T_{air}\right)\;\mathrm{for}\;\left(L_{T}-L_{T,flat}\right)<x<L_{T}\;,\;-W_{T}/2<y<W_{T}/2 \end{array}$$

(11) $$\begin{array}{cc} \; & \pm \left(k_{x}\frac{\partial T}{\partial x}+k_{y}\frac{\partial T}{\partial y}\right)=q_{ir}+h_{R}\left(T-T_{roller}\right)\;\mathrm{for}\;0<x<L_{T,flat}\;,\;-W_{T}/2<y<W_{T}/2 \end{array}$$

(12) $$\begin{array}{cc} \; & \frac{\partial T}{\partial x}=0\;\mathrm{at}\;x=0 \end{array}$$

(13) $$\begin{array}{cc} \; & T=T_{incoming}\;\mathrm{at}\;x=L_{T} \end{array}$$

(14) $$\begin{array}{cc} \; & \frac{\partial T}{\partial y}=0\;\mathrm{at}\;y=-W_{T}/2,W_{T}/2 \end{array}$$

Substrate (3D):

(15) $$\begin{array}{cc} \; & \pm k_{z}\frac{\partial T}{\partial z}=q_{ir}+h_{air}\left(T-T_{air}\right)\;\mathrm{at}\;z=0 \end{array}$$

(16) $$\begin{array}{cc} \; & \pm k_{z}\frac{\partial T}{\partial z}=h_{tooling}\left(T-T_{tooling}\right)\;\mathrm{at}\;z=th_{S} \end{array}$$

(17) $$\begin{array}{cc} \; & \frac{\partial T}{\partial x}=0\;\mathrm{at}\;x=0 \end{array}$$

(18) $$\begin{array}{cc} \; & T=T_{incoming}\;\mathrm{at}\;x=L_{S} \end{array}$$

(19) $$\begin{array}{cc} \; & \frac{\partial T}{\partial y}=0\;\mathrm{at}\;y=-W_{S}/2,W_{S}/2 \end{array}$$

where $q_{ir}$ was defined as the heat flux generated in the optical model considering the absorbed and reflected energies ($\phi_{i,a}$ and $\phi_{i,a,ref}$), $h_{air}$, $h_{tooling}$ and $h_{R}$ were the heat transfer coefficient of air, tooling (mandrel) and roller, respectively, $T_{air}$, $T_{tooling}$, $T_{roller}$ and $T_{incoming}$ were the air, tooling (mandrel), roller and incoming material temperature, respectively. The thermal model convergence was checked concerning the advection term used in the steady-state heat conduction equation. Based on the preliminary convergence study, the total number of control volumes was determined as 600 (40 and 15 in the *x*- and *y*-direction, respectively) for the tape and 9000 (40, 15 and 15 in the *x*-, *y*- and *z*-direction, respectively) for the substrate.

**Figure 7:**
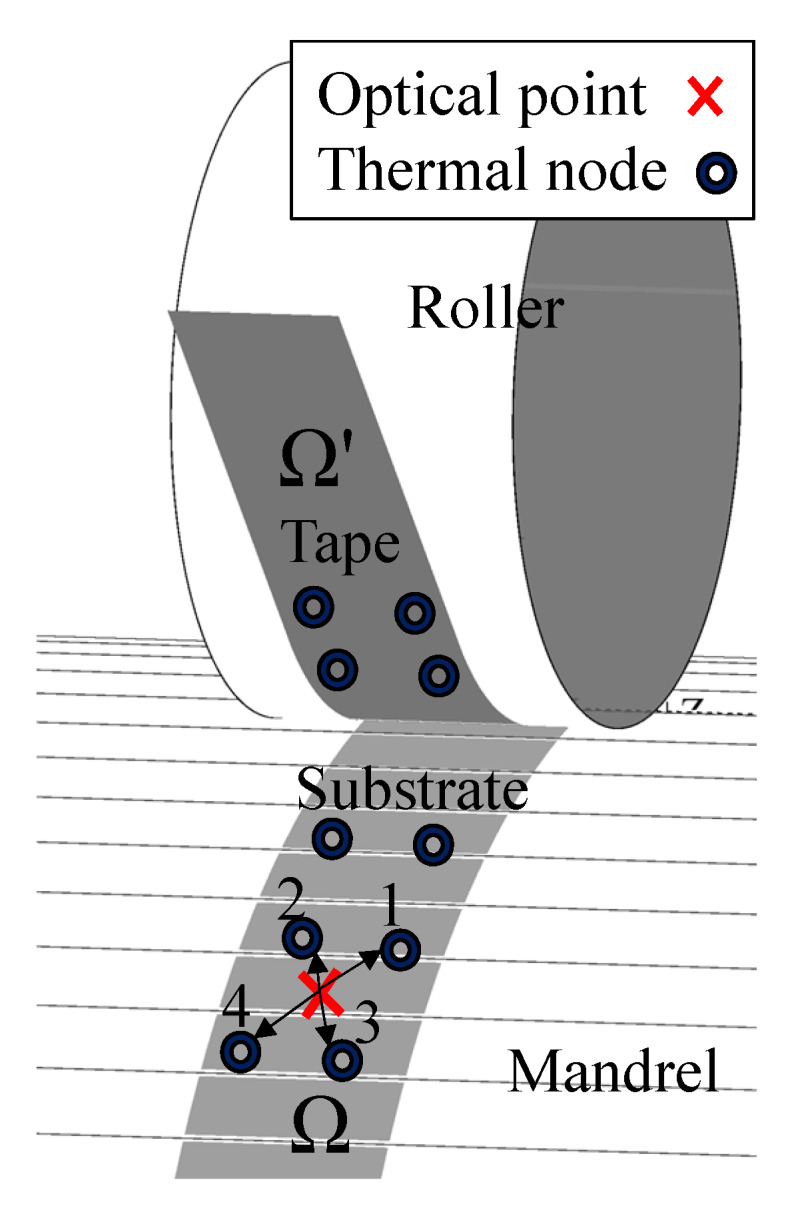
Distribution of the exemplary absorbed energy ($\phi_{i}$) inside the thermal domains (Ω for the substrate, $\Omega^{\prime }$ for the tape).

**Figure 8:**
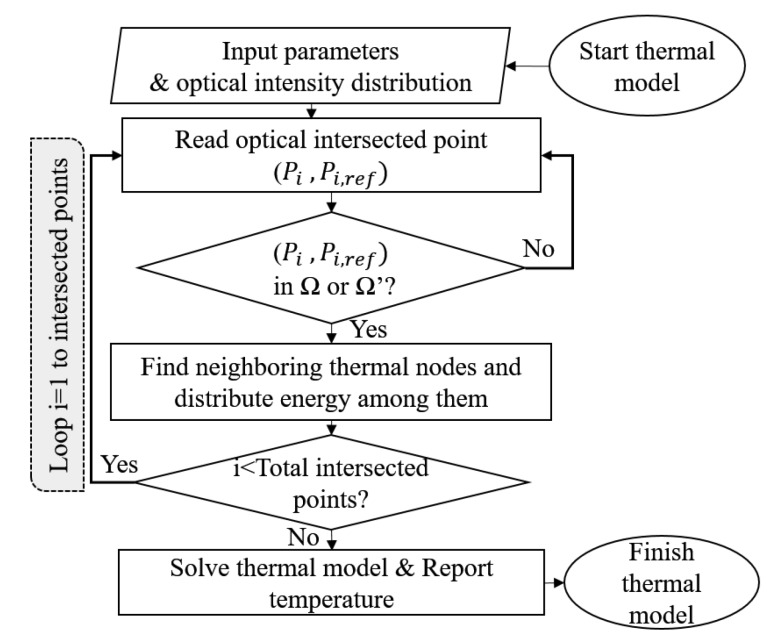
Flowchart of the implemented thermal model.

Since the number of rays defined in the optical model was larger than the control volumes defined on the tape and substrate surface (at z=0), a mapping procedure was defined in order to translate the laser irradiation from optical points to the control volumes in the thermal model. The energy of each incoming ray defined in the optical model was distributed among the four closest control volumes in the thermal model based on the distance between the intersection point of the ray and corresponding control volume using the expression in Equations (20) and (21):

(20) $$\begin{array}{c} w_{j}=\frac{\frac{1}{d_{j}}}{\frac{1}{d_{1}}+\frac{1}{d_{2}}+\frac{1}{d_{3}}+\frac{1}{d_{4}}},\;\left(j=1,2,3,4\right) \end{array}$$

(21) $$\begin{array}{c} \phi_{i}=w_{1}\phi_{i}+w_{2}\phi_{i}+w_{3}\phi_{i}+w_{4}\phi_{i}\;\;\;\; \end{array}$$

where $d_{j}$ was the distance between the intersection point of the *i*th ray and *j*th control volume within the closest four control volumes, $w_{j}$ was the corresponding weighting function and $\phi_{i}$ was the total absorbed energy by the tape or substrate. This procedure is schematically illustrated in Figure 7 and was carried out for the whole tape and substrate domains. The flowchart of the described thermal model in this section is represented in Figure 8.

## 4. Model Parameters and Case Studies

The developed optical-thermal model was utilized to simulate the LATP process of CF/PEEK composites reported in References [11,24] for verifying the numerical implementation. Subsequently, various case studies for the LATP and helical LATW processes were performed. In LATW processes, usually, the linear velocity is incorporated which is translated from the angular velocity and the lateral movement of the laser head. Therefore, a linear velocity was assumed for all the cases in this work. The geometrical parameters used in the process simulations are listed in Table 1. The corresponding material and process parameters are given in Table 2. The unidirectional GF/HDPE (glass fiber high density polyethylene) thermoplastic tape [33] was employed in the present work aligning with the EU funded ambliFibre project objectives with the focus on manufacturing low-cost thermoplastic products. The composite properties were estimated using a rule of mixture [34] based on the fiber volume content of the GF/HDPE which was 47% [33]. Note that the average values of the temperature dependent material data (ρ, $C_{p}$, $k_{x}$, $k_{y}$ and $k_{z}$) provided in References [11,24] were used in the present work for the model validation.

In order to study the effect of curved surface geometries and θ on the temperature distributions, different case studies were carried out using the model parameters listed in Table 3. It is seen that three different tape/substrate sizes (denoted as Ref, Wide-T and Narrow-T) were considered with three different mandrel sizes (denoted as Ref, Big-M and Small-M) for the helical LATW process. A total of five winding/placement angles was used for each case. Only θ = 0° was considered for the LATP process with three different tape/substrate sizes. The total number of process simulations performed in the case studies was 28.

## 5. Result and Discussion

### 5.1. Process Model Validation

**Figure 9:**
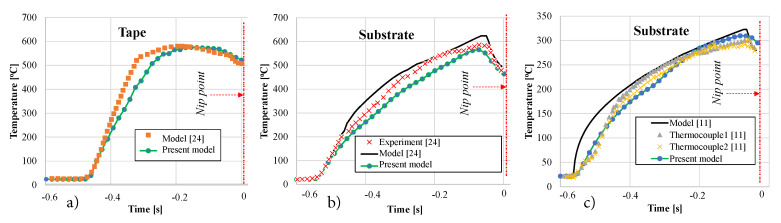
Tape (**a**) and substrate (**b**,**c**) temperature results from Reference [11,24] and corresponding results from the developed present simulation model. The temperature of a particle through the time from laser irradiation until nip point is shown.

The tape and substrate temperature distributions prior to the nip line/point were predicted using the proposed optical-thermal model for the LATP process reported in References [11,24] and the results are shown in Figure 9. It is seen from Figure 9 that the predicted temperature distributions at the centerline of the tape and substrate along the winding/placement direction agreed well with the reported ones in References [11,24]. The discrepancy between the predicted and reported tape temperatures was due to the differently implemented modelling approaches. More specifically, the tape was modelled as a 2D domain considering the *x*-*z* plane using the finite element method in Reference [11], whereas the *x*-*y* plane seen in Figure 6 was employed with a control volume based finite difference approach in the present work. Another source of tape temperature difference, which was also valid for the substrate, was due to the constant thermal material properties in the current model and temperature-dependent one in Reference [24]. However, both tape models resulted in very similar cooling rates and shadowed regions at the nip point with the temperature of approximately 500 °C–520 °C. The length of the shadowed regions on the substrate was found to be approximately 4 mm and 6 mm for the configurations reported in Reference [11] and Reference [24], respectively. The corresponding predicted substrate temperatures at the nip point were found to be approximately 470 °C and 300 °C. Although a 2D computational domain, that is, *x*-*z* plane seen in Figure 6, was used for the substrate in References [11,24], similar laser irradiation lengths and temperature evolutions were obtained using the 3D optical-thermal model for the substrate. The details of the optical model output are discussed in detail in the following.

### 5.2. Case Studies

#### 5.2.1. Optical Analysis

**Figure 10:**
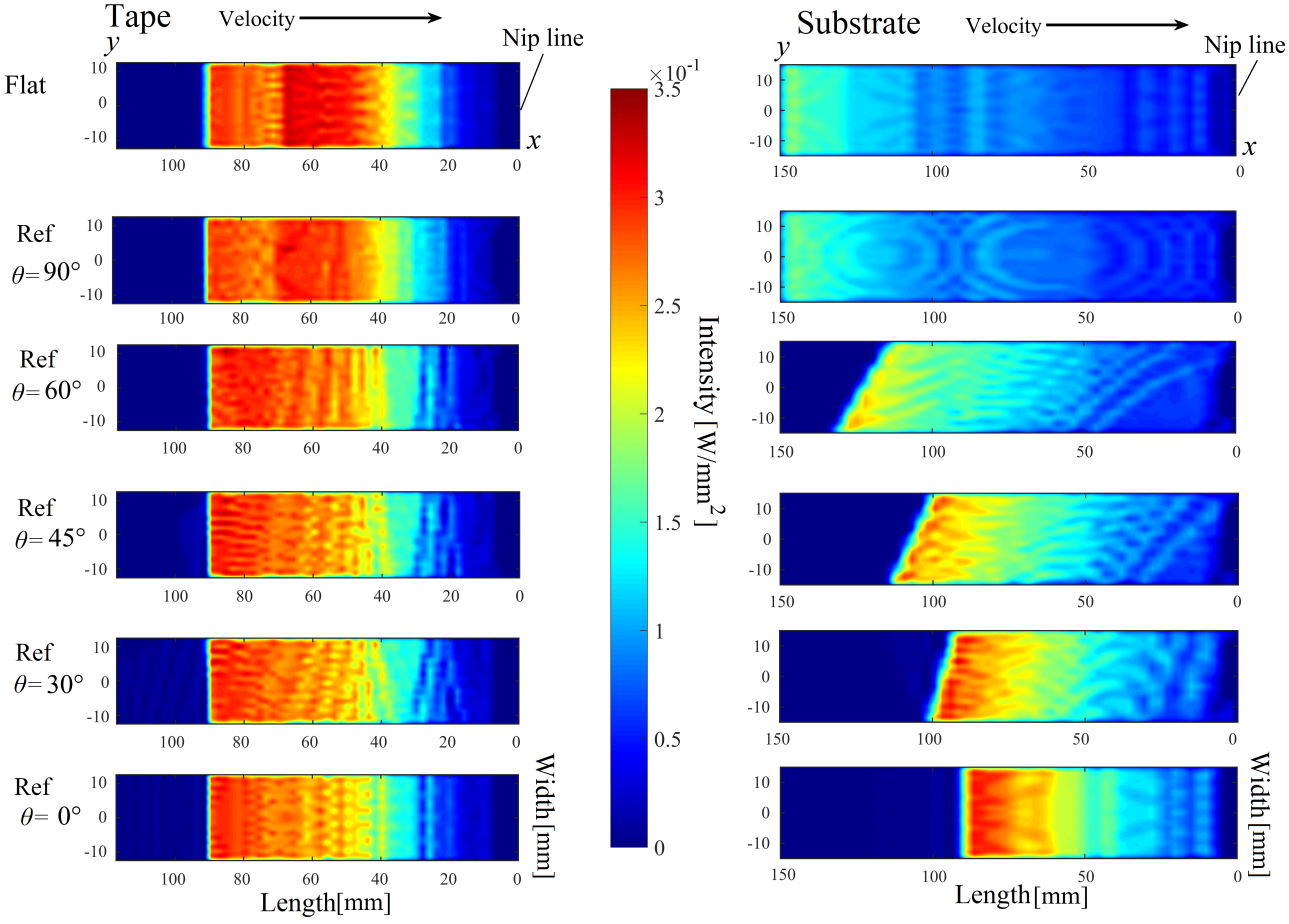
Absorbed power intensity distribution on the tape and the substrate for the cases Flat and Ref ($W_{T}=25$ mm and $W_{S}=30$ mm) using different θ. (For interpretation of the references to color in this figure legend, the reader is referred to the web version of this article).

The main outcome of the 3D optical model was the absorbed power intensity distribution on the tape and substrate. The obtained distributions for the cases Flat and Ref using different θ are shown in Figure 10 by unfolding the respective 3D surfaces into 2D ones. The slight scatter in the intensity distribution in Figure 10 was mainly due to the utilized finite number of laser rays that originated from the discretized laser source. Nevertheless, the sufficient number of rays were used in the optical model which provided converged temperature output in the thermal model in which the finite difference method was employed. It is seen in Figure 10 that the intensity distributions on the tape surfaces were almost identical for different winding/placement configurations which was as expected because the laser orientation was in parallel with the tape and the tape geometry remained constant for all simulations. The slight differences, for example, for θ=30°,45°,60°, were due to the reflections from the substrate. The total irradiation length of the tape was approximately 60 mm and the region 20 mm prior to the nip line was not heated, that is, the so-called shadow area, due to the curvature of the tape that is in contact with the roller as seen in Figure 2. The effect of the mandrel curvature on the intensity distribution was found to be more significant for the substrate. A more non-uniform and non-symmetric intensity distribution was predicted especially for θ=30°,45°,60° due to more complex laser irradiation and reflections. The shadow region for the substrate was found to occupy a smaller area as compared with the shadow regions for the tape. In addition, the reflections coming from the roller as well as the laser divergence also played a role on the intensity distribution on the substrate as can be understood from the slight reduction in the intensity magnitude at the edges, that is, at y=−12.5 mm and y=12.5 mm (see Figure 10). The length of the heated region reduced approximately from 140 mm to 75 mm as θ reduced from 90° to 0°, as expected from Figure 4. On the other hand the magnitude of the intensity increased with a decrease in θ due to an increase in the incident angle β. The effect of the laser divergence angle γ can be seen for Flat and Ref-90° cases in which the laser intensity magnitude was slightly smaller near the nip line region than the region far away from the nip line. A gradual decrease in the intensity magnitude was found towards the nip line for θ=0°,30°,45°,60° mainly due to a decrease in β. The reflections on the substrate surface coming from the tape and roller were also visible, for example, the region approximately 50–60 mm away from the nip line for Flat and Ref-90° cases. The maximum laser intensity value on the substrate was calculated as 0.35 W/mm${}^{2}$ for θ=0°.

**Figure 11:**
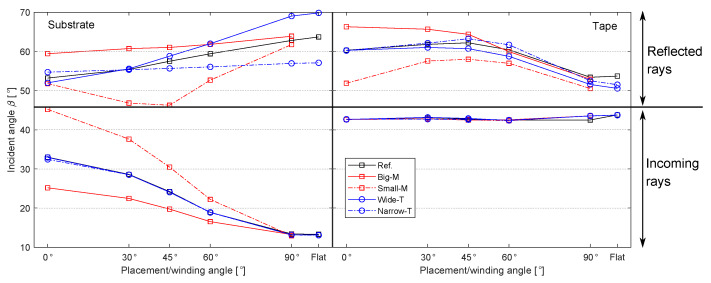
The mean of β distribution on the substrate and tape surfaces for each case study listed in Table 3.

**Figure 12:**
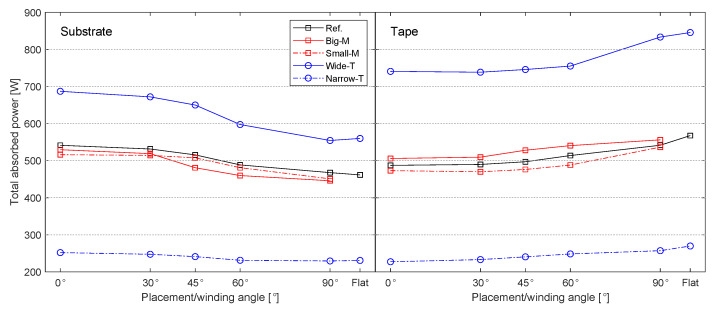
The total absorbed power for the substrate and tape calculated based on the incoming and reflected energies for each case study listed in Table 3.

**Figure 13:**
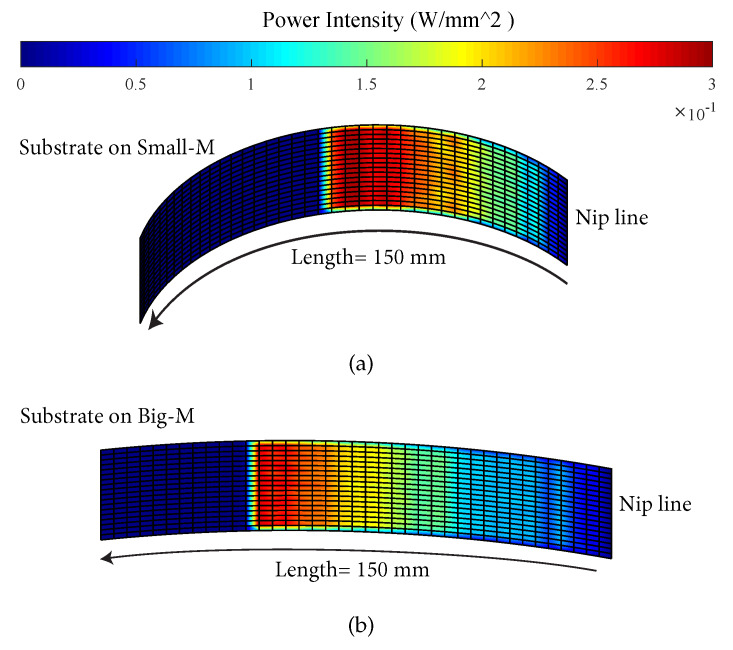
Effect of substrate curvature due to the mandrel diameter on the laser irradiated area and its absorbed power intensity on the case Ref-0° for: (**a**) Small-M, (**b**) Big-M (For interpretation of the references to color in this figure legend, the reader is referred to the web version of this article).

The heat flux distributions on the substrate and tape surfaces were determined by the incident angle β of the incoming and reflected rays as well as by the illuminated (and heated) area by the incoming and reflected rays. The mean of β for each case study listed in Table 3 was calculated using the optical model and the obtained results are shown in Figure 11. It is seen that the mean of β is always smaller than 45° for the incoming rays and the mean of β is always larger than 45° for the reflected rays for both tape and substrate. It was found that β for the incoming rays of the substrate gradually decreased from a range of 25°–45° to a constant value of 14° with an increase in θ from 0° to 90°, mainly due to the change in the surface curvature. The decrease in β was the most significant for the case Small-M due to a greater curvature, leading to much higher values of β. On the other hand, β of the incoming rays on the tape remained almost the same at approximately 42° since the tape geometry was not changed with θ. In addition, the incoming rays with relatively high β values covered a larger area on the tape than the substrate. This resulted in a larger heated area on the tape surface with higher laser intensity distribution which can be seen from Figure 10 as well. There was a gradual increase in the mean of β for the reflected rays of the substrate coming from the tape and roller with an increase in θ due to the fact that a larger amount of reflected rays hit the substrate surface. Similarly, the tape received larger amounts of reflected rays as θ decreased because a larger amount of rays reflected towards the ambient environment and not to the tape when θ decreased. This can also be seen from the heat flux distribution for the case Ref displayed in Figure 10 such that at θ = 90° locally a higher intensity is approximately between 60–70 mm before the nip line as compared with 0° for the tape. The corresponding total absorbed powers by the substrate and tape for each case are shown in Figure 12. It is seen that the total power absorbed by the substrate gradually decreases with an increase in θ mainly due to a reduction in the mean β. On the other hand, this trend was found to be the opposite for the tape, although the mean of β remained the same. The reason behind this was the increase in the intensity of illuminated or heated area for the tape by the number of reflected rays coming from the substrate as θ increased. The case Wide-T had the largest absorbed total power for the substrate and tape since the heated area was the largest for this case. The influence of the mandrel size on the power intensity distribution of the substrate is illustrated in Figure 13. It is seen that a more localized power intensity distribution occurred for the Small-M due to higher laser incident angles on the substrate. This localized behavior on the Small-M which took place near the nip line caused a smaller heat loss from the irradiation location until the nip line via the convection than the corresponding heat loss of the Big-M.

**Figure 14:**
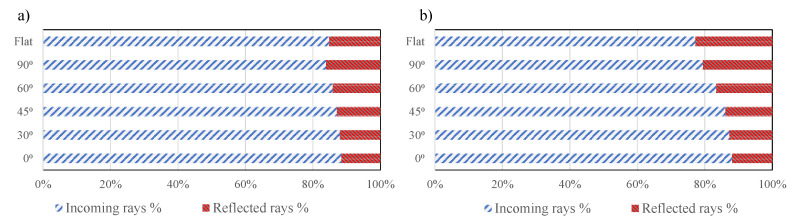
The distribution of the total absorbed power based on the incoming and reflected rays of (**a**) substrate and (**b**) tape for case Ref and Flat with different θ.

The overview of the corresponding contribution for the total absorbed power from the incoming and reflected rays is shown in Figure 14 for the case Ref and Flat as an example. It is seen that up to 20% of the absorbed power was from the reflected rays for the tape with θ = 90° and it was approximately 12% for the θ = 0°. It was even slightly more for the Flat case than for Ref-90° as the substrate surface gradient was more toward the tape. The absorbed power by the substrate from the reflected rays was approximately 12–18%.

#### 5.2.2. Thermal Analysis

**Figure 15:**
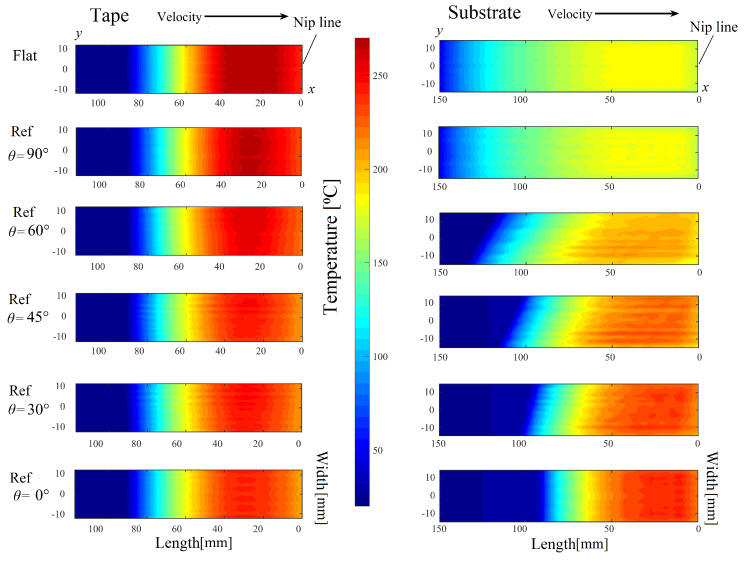
Predicted temperature distribution on the tape and the substrate for the cases Flat and Ref ($W_{T}=25$ mm and $W_{S}=30$ mm) using different θ. (For interpretation of the references to color in this figure legend, the reader is referred to the web version of this article).

The predicted temperature distributions on the tape and substrate surfaces are shown in Figure 15 for the case Ref and Flat. The more localized absorbed laser intensity on the tape due to the reflections from the substrate resulted in higher temperature values which can be seen in Figure 15 for the cases Flat, Ref-90° and Ref-60°. The substrate temperatures increased with a decrease in θ due to an increase in the laser incident angle and stronger light absorption. On the other hand, the tape temperature decreased with a decrease in θ because less reflections from the substrate to the tape took place for lower θ values with respect to the winding/placement curvature. The temperature at the nip line was found to vary between 200 °C–250 °C for the tape and 160 °C–220 °C for the substrate. The higher tape temperature as compared with the substrate was mainly due to the higher incident angle of the tape (see Figure 11) as also discussed in Section 5.2.1.

**Figure 16:**
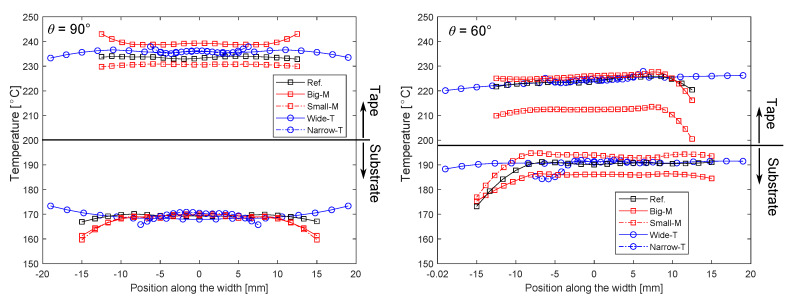
Temperature distribution at the nip line for each case at θ = 90° (**left**) and 60° (**right**). (For interpretation of the references to color in this figure legend, the reader is referred to the web version of this article).

The temperature distributions at the nip line were further investigated in detail for each case study since the nip line temperature played an important role on the bonding quality of the tape and substrate as aforementioned. To illustrate, the temperature distribution along the nip line is shown in Figure 16 for each case with θ = 90° and θ = 60°. It is seen that there is a temperature variation along the nip line for the tape and substrate mainly due to mandrel curvature, laser divergence, laser reflections from tape and roller, that is, the tape had a larger refractive index than the roller. This effect can be easily distinguished for the cases Narrow-T and Wide-T where Narrow-T had more reflections from the roller than Wide-T.

**Figure 17:**
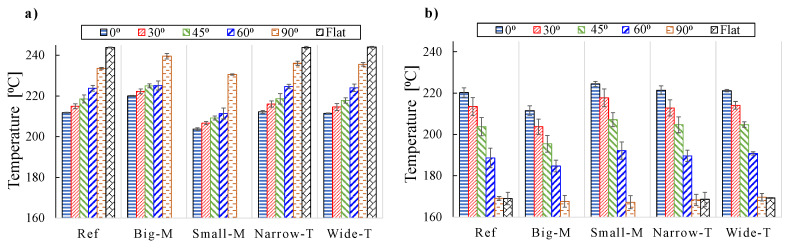
Average temperatures of (**a**) tape and (**b**) substrate and corresponding standard deviations at the nip line for each simulation case as a function of selected θ values.

The effects of reflections from the roller on the substrate were observed from the temperature distributions for Wide-T and Narrow-T at θ = 90°, that is, the substrate temperatures at the edges were lower for Narrow-T than Wide-T due to a smaller amount of reflected energy from the roller in the Narrow-T case. Therefore, the Wide-T case yielded higher substrate temperatures with an amount of approximately 5 °C increase at the edges of the substrate. The influence of θ on the temperature variation at the nip line was more significant which resulted in a reduction in the temperature at one of the substrate/tape edges by approximately 20 °C as seen in Figure 16 (right). The mandrel curvature affected the nip line temperature variation by approximately 15 °C for θ = 60°. The means and standard deviations of the temperature variations at the nip line are summarized in Figure 17. It is seen that the Big-M case resulted in higher tape temperatures than the Small-M case which was due to a stronger reflection contribution from the substrate to the tape. The other way around was the case for the substrate, that is, lower temperatures were obtained for the Big-M case as compared with the Small-M case due to smaller laser incident angles for the larger mandrel. The effect of θ on the mean nip line temperature can be easily seen in Figure 17 for the tape and substrate. The standard deviation of the substrate temperature was found to be higher than of the tape which was due to the mandrel curvature. The largest standard deviation of the substrate temperature distribution was approximately 4.7 °C for θ = 60° for the case Ref which resulted in a coefficient of variation of 2.5%. The corresponding maximum coefficient of variation was approximately 1.3% for the tape temperature obtained for θ = 60° of the Small-M.

The emerged tape and substrate temperature variation along the nip line especially for θ = 30°, 45° and 60° should be kept between certain material-dependent upper/lower temperature bounds in order to have proper consolidation quality which can be controlled by an optimized laser intensity. The temperature variations based on θ, mandrel size and tape width as summarized in Figure 17 are very critical since an overheating might take place for the tape on a higher θ and a low-melting temperature at the same time might be the case for the substrate. This is a challenge for the power intensity distribution as the laser source should provide a controllable energy distribution between the tape and substrate for different process conditions. Optimizing the laser settings such as the laser direction, the laser location or the laser distribution with the goal of desired temperature distribution is therefore essential to keep the tape and substrate temperatures at the desired levels.

## 6. Conclusions

In this research, a generic combined optical-thermal model of the LATP/LATW processes was presented to predict the temperature development of the tape and the substrate for helical paths on cylindrically shaped mandrels. The developed optical-thermal model was first validated successfully with available literature data of the LATP on flat tooling. Subsequently, the influences of the winding/placement angle, mandrel radius and tape/substrate width on the absorbed laser intensity and the corresponding temperature distributions on the tape and the substrate were investigated. The relations between the incident angle of the incoming/reflected laser rays and total absorbed absorbed power were quantified using the optical model. The temperature distributions on the substrate and tape surfaces as well as on the nip line were analyzed critically using the thermal model. The variations in temperature with respect to different winding/placement angles, mandrel sizes and tape widths were analyzed systematically. The following conclusions from the validated model can be drawn:

An increase in the winding/placement angle resulted in higher temperatures for the tape and lower ones for the substrate. The increase in tape temperatures was approximately 30°C–40°C and decrease in substrate temperatures was approximately 40°C–50°C. More contribution from reflections from the substrate for higher θ values resulted in higher tape temperatures. On the other hand, higher substrate temperatures were obtained with larger incoming laser incident angles for lower θ values. Similarly, higher substrate temperatures were the case for the mandrel with relatively small diameter (Small-M) due to larger incident angles. A reduction in tape temperature was found for Small-M case because some of the reflections from the substrate did not illuminate the tape. The tape and substrate temperatures were found to be less sensitive to a change in tape/substrate width as compared with the winding/placement angle and the mandrel diameter. However, the tape/substrate width effect on the tape and substrate temperature was manifested through a crescent temperature profile along the nip line where temperature variations occurred mainly at the edges of the tape and substrate.

The detailed analysis conducted in this paper showed the importance of the relation between the mandrel surface curvature and tape/substrate temperature for different geometrical configurations of the processing zone. The demonstrated opposing behavior of the total absorbed power and nip line temperature of the tape and substrate is very important especially in order to prevent temperature variations, overheating or having too low temperatures for either tape or substrate before the bonding of the tape onto the substrate at the nip line. One of the potential solutions to minimize the temperature variation at the nip line is the optimization of the laser location, orientation and/or laser distribution with respect to different winding/placement angles and different geometrical configurations. This is part of currently ongoing work.

## Figures and Tables

**Table 1:** Geometrical parameters used in the current modeling work and model validation based on the available data in References [11,24] according to the schematic geometrical model domain given in Figure 2.

Geometrical Parameters	Symbol	LATW/LATP Current Work (Reference Case)	LATP [11] (Model Validation)	LATP [24] (Model Validation)	Unit
Laser location(X,Y)	$P_{L}$	200, 16	200, 84	171.6, 98.1	[mm]
Laser source width	$W_{L}$	40	16 *	16 **	[mm]
Laser source height	$H_{L}$	50	45 *	45 **	[mm]
Laser source angle	$\theta_{L}$	11.8	22.7	32	[°]
Laser divergence angle	γ	4 [30]	N/A	3	[°]
Tape width	$W_{T}$	25 [33]	12	12	[mm]
Tape thickness	$th_{T}$	0.25 [33]	0.15	0.15	[mm]
Roller radius	$R_{R}$	45	32	40	[mm]
Roller width	$W_{R}$	50	50	40	[mm]
Length of flat part of tape	$L_{T,flat}$	78	55	50	[mm]
Length of tape-roller contact	$\theta_{R}$	50.0	50	67.4	[°]
Substrate length	$L_{S}$	150	120	90	[mm]
Substrate width	$W_{S}$	30	30	25	[mm]
Substrate thickness	$th_{S}$	0.25	3.48	1.65	[mm]
Mandrel radius	$R_{M}$	136	*∞*	*∞*	[mm]
Winding/placement angle	θ	0–90	0	0	[°]

* at distance of 127 mm from the laser source aperture, ** at distance of 200 mm from the laser source aperture.

**Table 2:** Process and material properties used in the current modeling work and model validation based on the available data in References [11,24].

Process/Material Parameters	Symbol	GF/HDPE (Current Work)	CF/PEEK [11] (Model Validation)	CF/PEEK [24] (Model Validation)	Unit
Composite thermal conductivity	$k_{x}$,$k_{y}$,$k_{z}$	0.85, 0.65, 0.65 [33]	5.9, 0.7, 0.7	5.9, 0.7, 0.7	[W/(m-K)]
Composite density	ρ	1750.0 [33]	1575	1575	[kg/m^3^]
Composite specific heat	$C_{p}$	1220.0 [33]	1500	1500	[J/(kg-K)]
Winding/placement linear velocity	*v*	100	133	100	[mm/s]
Total laser power	$P_{laser}$	2500	1100 (assumed)	1130	[W]
Air heat transfer coefficient	$h_{air}$	10 [6]	10	10	[W/(m^2^-K]
Air temperature	$T_{air}$	20	20	20	[°C]
Roller heat transfer coefficient	$h_{R}$	100 [6]	500	500	[W/(m^2^-K]
Roller temperature	$T_{roller}$	20	100	50	[°C]
Tooling heat transfer coefficient	$h_{tooling}$	100 [6]	*∞*	*∞*	[W/(m^2^-K]
Tooling temperature	$T_{tooling}$	20	20	20	[°C]
Incoming material temperature	$T_{incoming}$	20	20	20	[°C]
Composite refractive index	$n_{c}$	1.95 [24]	1.95	1.95	-
Roller refractive index	$n_{R}$	1.43 [24]	1.43	1.43	-

**Table 3:** Summary of the case studies performed using the developed parametric optical-thermal model.

Case Studies	$R_{M}$ [mm]	$W_{T}$ [mm]	$W_{S}$ [mm]	θ [°]	Total Simulations
Flat	*∞*	12.5, 25, 38	15, 30, 38	0	3
Reference (Ref)	136	25	30	0, 30, 45, 60, 90	5
Big mandrel (Big-M)	272	25	30	0, 30, 45, 60, 90	5
Small mandrel (Small-M)	68	25	30	0, 30, 45, 60, 90	5
Wide tape (Wide-T)	136	38	38	0, 30, 45, 60, 90	5
Narrow tape (Narrow-T)	136	12.5	15	0, 30, 45, 60, 90	5
